# Osteosynthesis and outcomes of traumatic periprosthetic femoral fractures after total hip arthroplasty

**DOI:** 10.1186/s42836-021-00089-1

**Published:** 2021-10-01

**Authors:** Ali Taha, ElZaher Hassan ElZaher, Ibrahim ElGanzoury, Mostafa Ashoub, Amr Khairy

**Affiliations:** grid.488444.0 0000 0004 0621 8000 Ain, Shams University Hospital, Cairo, Egypt

**Keywords:** Periprosthetic femoral fracture, Osteosynthesis, Hip, Arthroplasty, Fracture

## Abstract

**Purpose:**

The aim of this retrospective study was to investigate the treatment of traumatic periprosthetic femoral fractures with open reduction and internal fixation. The outcomes with the use of the surgical techniques were also reported.

**Methods:**

Between September 2017 and September 2019, 25 patients with traumatic periprosthetic femoral fractures were managed by open reduction and internal fixation in Ain Shams University Hospital, Egypt. The fixation methods were selected based on the surgeon’s preference. Outcomes were assessed using the Harris Hip Score, visual analogue score (VAS) for pain, and EuroQol 5 Dimensions – 5 Level (EQ5D-5L) prior to and after surgery. Patients were regularly followed up for one year. A *P* value < 0.05 was considered to be statistically significant.

**Results:**

The mean age at surgery was 77 years (range, 51 to 95 years), 64% (*n* = *16)* were females. According to the Vancouver classification, there were 1 type AG, 15 type B1, and 9 type C fractures. Postoperative complications included wound site infection (*n* = 2) and non-union (*n* = 1). The mean pre-trauma Harris Hip Score was 77.44 ± 8.63 (range, 65 to 90), and the mean Harris Hip Score collected at the final follow-up was 72.47 ± 8.85 (range, 60 to 86) (*P* < 0.05). The mean pre-trauma VAS was 2.20 ± 1.21 (range, 0 to 4), and the mean VAS recorded at the final follow-up was 3.00 ± 1.41 (range, 0 to 5) (*P* < 0.05). According to the EQ5D-DL assessed at the final follow-up, no patient felt that their daily life and activities became more problematic.

**Conclusion:**

This study provided added validation of the current management of periprosthetic femoral fractures after total hip arthroplasty. Using the proper fixation and implant can achieve a reliable fixation and good functional recovery.

**Level of evidence:**

IVa

## Background

Periprosthetic femoral fractures (PFFs) are one of the complications after hip arthroplasties. The reported incidences of PPF after primary total hip arthroplasty (THA) ranged from 0.1 to 18% [1]. It is estimated that the number of THA has increased by almost 30% in many countries between 2007 and 2017 [2]. It causes a sequential increase in the rate of PFFs [3, 4]. Currently, managing these fractures is still challenging.

The treatments are based on the fracture characteristics, such as location, implant stability, fracture pattern, and quality of bone stock [5, 6]. The Vancouver classification offers a reproducible description of these factors with the subsequently easy formation of a treatment plan (which facilitates treatment planning) [7]. Various treatment options are available for PFFs. Non-operative treatments include traction or the use of a spica cast or cast brace [8]. However, the treatments are often associated with high rates of complications, such as prosthetic loosening, malunion, non-union, skin ulceration, deep venous thrombosis, and other medical problems [9]. Surgical strategies are selected based on those fracture characteristics [10]. Operations involve the minimally invasive procedures and conventional open reduction and internal fixation, with or without bone grafting [11, 12].

With patients with femoral component loosening, a revision operation is usually recommended. The procedures include replacement with a longer stem that provides more intramedullary stabilization. It can be done with or without the help of extramedullary allograft supplementation [10]. It is only indicated in un-displaced trochanteric fractures (type A) or when the patient’s medical condition precludes surgery [13].

With other fracture types, open reduction and internal fixation is a good choice. The implants include cerclage wires [9], dynamic compression plates [14–17], Mennen plates, Ogden plates, Partridge nylon plates, and straps [18–21]. Cable-plate systems [22–25] are selected for type B1 PFFs. Cortical on-lay allografts can be used as needed [16–18]. Retrograde intramedullary nailing is recommended for type C fractures that extend more distally.

This retrospective study aimed to investigate the treatment of PFFs with open reduction and internal fixation. We also reported the outcomes with the use of surgical techniques and implants.

## Materials and methods

This study involved human participants and was conducted in accordance with the ethical standards of the institutional and/or national research committee and with the 1964 Helsinki declaration and its later amendments or comparable ethical standards.

### Patient selection

We retrospectively reviewed 30 patients who presented with PFFs. The PFFs were managed by open reduction and internal fixation in the Trauma and Orthopaedic Department between September 2017 and September 2019. The inclusion criteria of the study included: (1) patients aged 18 years or above; (2) PPFs following primary cemented or uncemented THAs; (3) either single or both hips involved; (4) combined stem loosening or loss of bone stock. The exclusion criteria were: (1) patients younger than 18 years (having an immature skeleton); (2) abnormal mental capacity due to cognitive comorbidities; (3) patients who were unable or unwilling to provide consent; (4) patients who were unable to come for regular follow-up visits for any irremissible reasons (*n* = 3); (5) patients who died before the final follow-up visit (*n* = 2); (6) female patients in child-bearing age and planning to conceive within the study. Finally, 25 of 30 patients were recruited into this study.

According to the Vancouver classification [25], the PFF patients were divided into types A, B1, B2, B3, and C. Type A fractures occurred in the trochanteric area (type AG involving the greater and type AL involving the lesser trochanter); type B fractures took place in the tip region of femoral component and was subclassified as B1 (well-fixed stem), B2 (loose stem), and B3 (loose stem with deficient bone stock) fractures. In types B1 and B2, the bone stock around the femoral component was adequate, while in type B3, the deficiency of bone stock developed due to severe comminution or osteolysis. Type C fractures were located distally and at the tip of the stem.

### Implants and fixation techniques

**Fig. 1:**
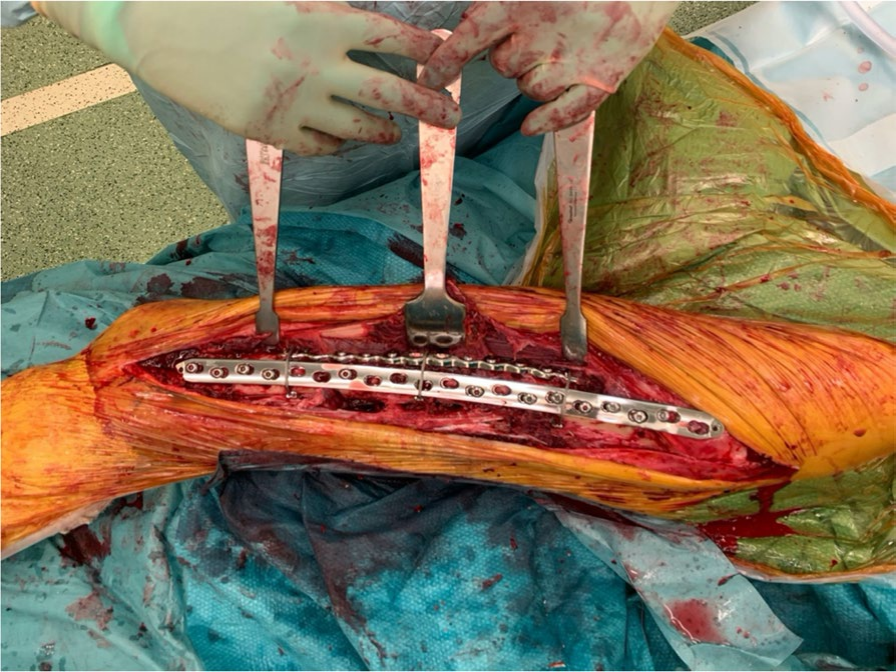
Using an additional cable plate to reinforce the fixation

**Fig. 2:**
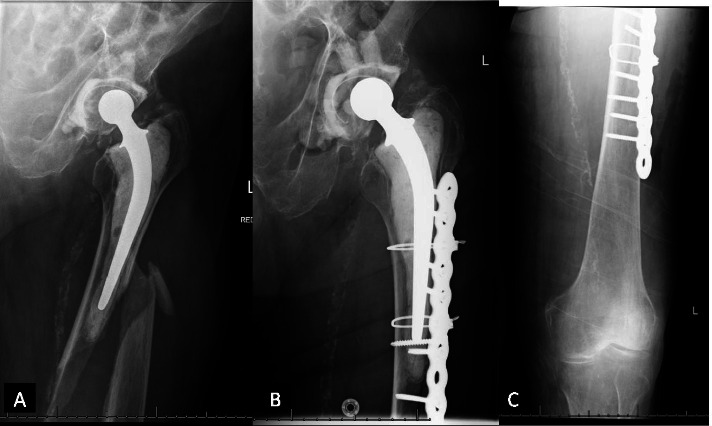
A periprosthetic femoral fracture (Vancouver type B1, left side). **A** Preoperative X-ray. **B** The X-ray taken 2 days after surgery shows the fracture is fixed with a cable plate system and locking screw. **C** The X-ray shows the middle portion of the femoral shaft

**Fig. 3:**
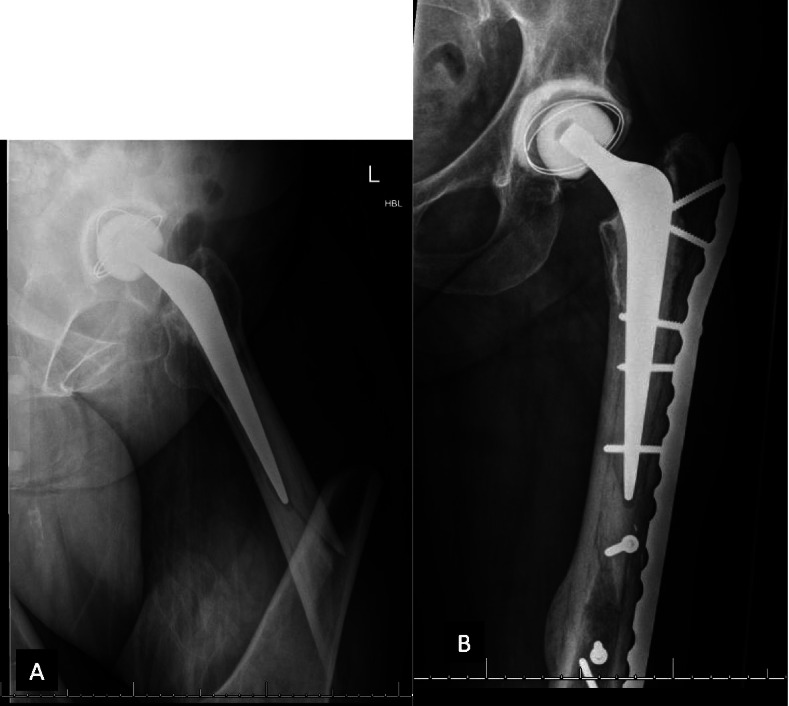
A periprosthetic femoral fracture. **A** Preoperative X-ray. **B** The fracture is fixed with a locking plate

**Fig. 4:**
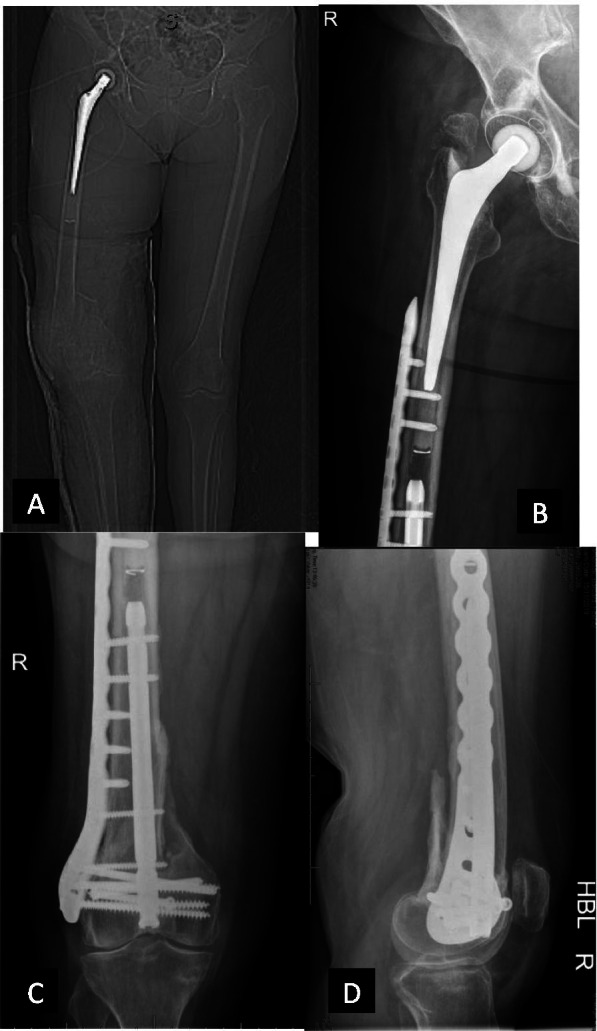
A periprosthetic femoral fracture (Vancouver type C). **A** Preoperative X-ray. **B** The fracture is fixed with an intramedullary nail. **C** Distal femur on anteroposterior X-ray. **D** Latera view

The implants used in this series included a double plate system (Fig. 1), cables, cable plate system (Fig. 2A, B, C), locking plate and screw system (Fig. 3A, B), and intramedullary nail. The implants were selected according to the surgeon’s preference and fracture types. The conventional open reduction and internal fixation was performed through the lateral incision on the hip and thigh. Retrograde intramedullary nailing was performed through the trans-patellar approach (Fig. 4A, B, C, D).

### Evaluation and analysis

X-rays were taken immediately after surgery and every 4 weeks thereafter until bone healing occurred. Bone healing was radiologically confirmed by the presence of callus formation across the fracture site. Hip pain was assessed in terms of the 10 cm visual analogue score (VAS) [26]. The function of the extremity was evaluated using the Harris Hip Score (HHS) [27]. We used the EQ5D-5L to measure patients’ health-related quality of life [28]. The *t*-test was utilized to determine the differences between the pre-trauma and postoperative data. A *P* value < 0.05 was considered to be statistically significant.

## Results

The demographics and surgical details of the patients are shown in Table 1. A total of 25 patients were included. There were 9 male and 16 female patients. The PFFs occurred at a mean time of 63 months (range, 6 to 120 months) after primary THA. According to the Vancouver classification, there were 1 type AG, 15 type B1, and 9 type C PFFs. The fixation techniques and assessments are detailed in Table 2. Postoperative wound infection occurred in 2 patients, which healed after debridement and wound care. Non-union occurred in one patient and was treated by a revision surgery 36 months after fracture fixation because her concomitant chronic cardiovascular diseases precluded an early revision surgery. This patient was excluded from the bone healing group. Neither cement fracture nor stem loosening was observed.

**Table 1:** Demographics and surgical details of 25 patients

Age (year; mean; range)	77 (51–95)
Gender (M: F)	9: 16
Side affected (L: R)	11: 14
Injury mechanism (*n*)	
Low energy	23 (92%)
Higher energy	2(8%)
Vancouver classification (*n*)	
Type AG	1 (4%)
Type B1	15 (60%)
Type C	9 (36%)
FOMAPA* (month; mean; range)	63 (6120)
Harris hip score	
Pre-trauma	77.44 ± 8.63 (65–90)
Final follow-up	72.47 ± 8.85 (60–86)
Difference	-4.970
*t*-test	6.935
*P* value (pre-trauma *vs.* postop)	< 0.001
VAS	
Pre-trauma	2.20 ± 1.21(0–4)
Final follow-up	3.00 ± 1.41(0–5)
Difference	0.8
*t*-test	-3.292
*P* value (pre-trauma *vs.* postop)	0.005
EQ 5D-5L at final follow-up (*n*)	
Mobility	
No problem (No change)	22 (92%)
Some problems	1 (4%)
A lot of problems	1 (4%)
Looking after oneself	
No problem (No change)	22 (92%)
Some problems	1 (4%)
A lot of problems	1 (4%)
Doing usual activities	
No problem (No change)	22 (92%)
Some problems	2 (8%)
A lot of problems	0
Pain	
No problem (No change)	21 (88%)
Some problems	2 (8%)
A lot of problems	1 (4%)
Feeling worried, sad or unhappy	
No problem (No change)	24 (100%)
Some problems	0
A lot of problems	0
Complications (*n*)	
Wound infection	2 (8%)
Non-union	1 (4%)
Bone healing (week; mean; range)**	22 (12–32)
Follow-up (month; mean; range)	18 (0–36)

FOMAPA*: Fracture Occurred *n* Months After Primary Arthroplasty; Bone Healing**, one case of non-union was excluded

**Table 2:** Detailed fixation techniques and assessments in 25 patients

	Cable plate	Plate	Tension band	IMN + Plate	Double plate	Total
Vancouver classification (n)						
A	0		1	0	0	1
B1	11	3	0	0	1	15
C	2	6	0	1	0	9
Total	13	9	1	1	1	25
Mean pre-trauma HHS						
A	0	0	69	0	0	69
B1	76	82	0	0	84	81
C	77	73	0	90	0	80
Mean of each fixation	77	78	69	90	84	77.44 ± 8.63
Mean HHS at final follow-up						
A	0	0	66	0	0	66
B1	71	78	0	0	80	76
C	65	69	0	82	0	72
Mean of each fixation	68	74	66	82	80	72.47 ± 8.85
Mean pre-trauma VAS						
A	0	0	3	0	0	3
B1	3	3	0	0	1	2
C	2	3	0	2	0	2
Mean of each fixation	3	3	3	0	1	2.2 ± 1.21
Mean VAS at final follow-up						
A	0	0	4	0	0	4
B1	4	4	0	0	2	3
C	2	2	0	3	0	2
Mean of each fixation	3	3	4	3	2	3 ± 1.41

Total data of assessment were recorded as mean ± standard deviation

A, B1, and C. Type A fractures occurred in the trochanteric area (type AG involving the greater and type AL involving the lesser trochanter); type B fractures took place in the tip region of femoral component and was subclassified as B1 (well-fixed stem), Type C fractures were located distally and at the tip of the stem

*IMN* intramedullary nail, *HHS* Harris Hip Score, *VAS* visual analogue score

The follow-up lasted for a mean of 18 months (range, 0 to 36 months). The mean pre-trauma HHS was 77.44 ± 8.63 (range, 65 to 90), and the mean HHS collected at the final follow-up was 72.47 ± 8.85 (range, 60 to 86) (*P* < 0.001). The mean pre-trauma VAS was 2.20 ± 1.21 (range, 0 to 4), and the mean VAS recorded at the final follow-up was 3.00 ± 1.41 (range, 0 to 5) (*P* = 0.005). According to the EQ5D-DL assessed at the final follow-up, no patient felt that his or her daily life and activities became more problematic, except for one patient who reported mild hip pain (Table 1).

## Discussion

PFF refers to any femoral fracture in a patient who has had a hip replacement [2]. The most common immediate cause of the fracture was a fall at home (66%) or outdoors (18%). The fractures are devastating complications that result in functional limitations, increase overall mortality, and pose great burdens on trauma and orthopaedic surgeons. The injury usually occurs in patients with multiple comorbidities, and the management tends to be difficult [29].

In this study, we used the validated method to classify the fracture patterns and then managed the fracture in a preferable way [30, 31]. The Vancouver classification is the guideline for the therapeutic planning. A successful treatment requires in-depth understanding of the nuances among fracture patterns, selecting and executing a rational treatment approach, and providing an appropriate postoperative recovery protocol. Unlike most other fractures, modification of standard techniques is often required.

In our experience, the surgical techniques and implants used in our study provided rigid fixation, resulting in a high-speed fracture healing, and most of the fractures healed with an acceptable mechanical alignment. Our study did not include types B2 and B3 fractures because these fractures are often associated with femoral component loosening that requires a revision THA [32]. The complication rates of PFF reportedly ranged from 26 to 43% [33, 34]. In our study, the complications included wound infection and non-union, but the incidence was lower [35].

Although functional recovery and rehabilitation usually takes a long period of time, especially in elderly patients, our study proved that the PFFs produce minimal disability after a proper treatment. Postoperative hip pain is rare. However, we could not predict when the fracture occurs and could not deliberately collect the data in advance. Therefore, the time for preoperative assessment was not unified.

Our study has some limitations. First, the sample size was relatively small due to the low incidence of PFFs. Second, most of our patients are elderly and consistent follow-ups were not possible. Third, the simultaneous degeneration changes of the lower limb are common in elderly patients, which might affect the outcomes of treatment. Fourth, many surgical techniques and implants were used in the study, and the options were based on the surgeon’s preference, which might produce a selection bias. Fifth, types B2 and B3 fractures were not included in this study, which might lead to an assessment bias. Sixth, the retrospective and unblinded design might lead to a statistical bias. Furthermore, future biomechanical investigations are needed to clarify the precise contribution of implant design to PFFs.

## Conclusion

This study provided added validation of the current management of PFFs after THA. Using the proper fixation and implant can achieve a reliable fixation and good functional recovery of the lower limb.

## Data Availability

Supporting data of this publication are available and will be furnished upon request.

## Abbreviations

EQ5D-5L: EuroQol 5 Dimensions – 5 Level
HHS: Harris Hip Score
IMN: Intra Medullary Nail
OECD: Organisation for Economic Co-operation and Development
ORIF: Open reduction and internal fixation
PPFX: Periprosthetic fractures
THR: Total hip replacement
VAS: Visual Analogue Score for Pain
